# The Case of a Lacerated Wound Which Penetrated the Thorax, from Which the Patient Recovered

**Published:** 1812-06

**Authors:** 

**Affiliations:** LEWISHAM


*For the Medical and Physical Journal.*

### THE CASE *of a* LACERATED WOUND *which penetrated the* THORAX, *from which the* PATIENT *recovered.*

BY MR. GREEN, LEWISHAM.

THE most uninformed medical student need not be told that large wounds which penetrate into the cavity of the thorax are highly dangerous, and most commonly end fatally; but the inference to be drawn from the following case is, that, however desperate and deplorable a wound may appear, yet we should never despair.

On Monday, the 9th of September, 1811, Edward Harpur, a strong hearty young man, aged 26, being at the baiting of a bull at Lewisham, and one of the foremost in the crowd encouraging the dogs, got unawares within the length of the confining rope: the bull instantly made at him, threw him down, and gored him. The tips of the horns of the animal had been sawed off, to prevent him from so readily wounding the dogs as he otherwise would have done; but this made it so much worse for the man. One of the horns struck him on the right breast just above the nipple, and tore through the integuments and pectoral muscles, and entered, between the fourth and fifth rib, into the thorax. The fourth rib was beat inwards, and the fifth was torn up from its connection with the sternum. A considerable portion of one of the right lobes of the lungs was protruding between the ribs; and the air was rushing with impetuosity through a large angular wound, into which I could put my hand, with its apex towards the neck.

The man was fainting repeatedly from pain, and appeared dying; and, when I was preparing to pass a ligature, or in common language to sew up the wound, the standers-by implored me to desist, and let him die quietly, and I heard a murmur that it was cruel to torment him more. I certainly had little hopes of the man's recovery; yet I thought it would appear more decent to dress the wound, and give him a chance, than let him remain in the mangled state he was in; and, having placed the ribs as near as possible in their proper situation, I passed a suture through the apex first, and brought the flap of integuments and muscles up. I then passed the needle through each side of the wound, and by this means closed, in a great measure, the aperture into the thorax. The man was in great torture all this time, but, as soon as the wound was dressed, the air no longer rushing impetuously through it, he became easier, and expressed great satisfaction. The reader may have some idea of the quantity of air that passed through the wound, when he is informed that the assistants were obliged to hold the candles at some distance on each side, for when held directly opposite the patient, they were near being extinguished. The accident happened about six o'clock, but he was bled from the arm, and some time had elapsed before I saw him. Ordered a cathartic mixture.

Tuesday, 10th. He passed the night tolerably quiet. Skin hot. Pulse about 96. No stool. The cathartic repeated. Ordered to be kept on a slight spare diet, and to have nothing to drink but barley water; in the evening an opiate.

Wednesday, 11th. A very restless night, great pain in the right shoulder, but none in the wound. The cathartic had procured five stools. The skin hot and dry. The tongue parched. The pulse nearly as yesterday. Ordered a powder with nitre and pulv. ipecac. comp. to be taken every six hours, and twelve ounces of blood to be taken from the arm.

Thursday, 12th. Seven in the morning. He has passed the night better than the last. The pain in the shoulder considerably abated. He has had two motions. Pulse 84. He thinks the bleeding relieved him very much. Ordered one dose of the cathartic mixture. The skin not so hot, and rather a moisture upon it. About four in the afternoon he became very hot and restless, and complained of great pain in the shoulder and wound: indeed he was universally ill. Venesection to fourteen ounces; and the anodyne at night.

Friday, 13th; seven in the morning. The cathartic had procured two motions; and he has had four hours comfortable sleep. Opened the wound for the first time; the fourth day from the accident. The right side of the angle had united by the first intention; the left side, or that nearest the sternum, did not appear closed. Removed the ligature from the right side; let the others remain. There is now, and has been from the second day, a great deal of emphysema over the breast. He complains of great pain in the chest, and, when considerable pressure is made with the whole hand, it gives him ease. This suggested the idea of confining the motion of the ribs; and I applied a double-headed roller round him, and pulled it tolerably tight, notwithstanding the wound, as we do for fractured ribs. Five grains of nitre and five of pulv. jalapii were prescribed for him every six hours. The skin, for the first time since the accident, feels cool. Pulse about 80, but not full or hard. In the evening of this day he complained of dreadful pain in his kidnies, and passed bloody urine. He finds no ease in any position but lying on his belly. Sixteen ounces of blood were taken from the arm, and an opiate given at night.

Saturday, 14th. He has passed a very bad night, in great pain in his back. He says he now would much rather die than pass such another night, for his pains are intolerable. Visited him at seven in the evening. He has slept some hours at intervals through the day. Dressed the wound, and removed the remaining ligatures. The crepitus or emphysematous feel totally gone.

Sunday, 15th. He has passed a tolerable night without the opiate. Pains all gone except a slight one in the chest. Skin cool. Pulse about 72. He has had three motions since yesterday. The wound looks well, and appears disposed to heal. Kept the angle up by means of adhesive plaister. Continue the powders.

Monday, 16th. Going on well; walks about his room.

Saturday, 20th. Still going on well. He this day walked

 out.

out. From this time not one bad symptom; but a considerable discharge, evidently from the inside of the thorax. He continued progressively getting better and better, and the discharge lessening every week; and by the 16th day of the ensuing month (October) he found himself capable of going to his work, which is that of a brick maker, and requires considerable exertions of the arms, which of course must call into action the pectoral and intercostal muscles.

October, 22d. The wound entirely healed, except a small opening about the size of a quil, into which a probe might be passed into a sinus which extended about three inches under the muscles towards the sternum, and from which an ugly fungus sprouted out. This fungus appeared to arise from one of the ribs: it was attached at the bottom of the sinus, but was loose, and protruding through the small external aperture. Its complexion was nearly black, and its substance parenchymatous, and insensible; for I repeatedly introduced the blades of a fine pair of scissars into the aperture, and cut it off, and sometimes took hold of it with a pair of forceps and tore it out, neither of which gave the smallest pain, but it bled considerably always after; and after it had been repeatedly destroyed by caustic (argent nitrat.) it as constantly sprouted out again. At length, after three months vexation, this fungus was at last destroyed, and the sinus healed, by injecting into it a strong solution of the hydrarg. muriatus; and I have the satisfaction of saying the man is now completely well and hearty.

It occurred to me that the most expeditious method of curing this man was dilating the sinus with the knife, and removing the fungus at once; but the patient could not bear to hear of it. He thought he had been wounded enough; and, while there remained a chance of his getting well without having recourse to the knife, he was contented to wait, and let every other method be tried first. It is worthy of remark, that after the second or third day the respiration was neither distressing or laborious; and I attribute the man's recovery principally to the repeated bleedings, and the small quantity of nutriment that was allowed him.

*March 13th,* 1812.

